# Gold Nanoparticles-MWCNT Based Aptasensor for Early Diagnosis of Prostate Cancer

**DOI:** 10.3390/bios12121130

**Published:** 2022-12-06

**Authors:** Aseel Alnaimi, Ammar Al-Hamry, Yahia Makableh, Anurag Adiraju, Olfa Kanoun

**Affiliations:** 1 Biomedical Engineering Department, Jordan University of Science and Technology, Irbid 22110, Jordan; 2 Professorship Measurement and Sensor Technology, Chemnitz University of Technology, 09111 Chemnitz, Germany; 3 Institute of Nanotechnology, Jordan University of Science and Technology, Irbid 22110, Jordan

**Keywords:** aptasensor, prostate cancer, prostate-specific antigen, carbon nanotubes, gold nanoparticles

## Abstract

Prostate cancer is one of the most frequently diagnosed male malignancies and can be detected by prostate-specific antigen (PSA) as a biomarker. To detect PSA, several studies have proposed using antibodies, which are not economical and require a long reaction time. In this study, we propose to use self-assembled thiolated single-strand DNA on electrodes functionalized by multi-walled carbon nanotubes (MWCNT) modified with gold nanoparticles (AuNPs) to realize a low-cost label-free electrochemical biosensor. In this regard, the PSA aptamer was immobilized via electrostatic adsorption on the surface of a screen-printed MWCNT/AuNPs electrode. The immobilization process was enhanced due to the presence of Au nanoparticles on the surface of the electrode. Surface characterization of the electrode at different stages of modification was performed by electrochemical impedance spectroscopy (EIS), atomic force microscopy (AFM) and Fourier transform infrared spectroscopy (FTIR) and contact angle for surface tension properties. The results showed an increase in surface roughness due to the absorbance of the aptamer on the electrode surfaces. The developed sensor has an extended linear range of 1–100 ng/mL, and a very low limit of detection down to 1 pg/mL. In addition, the reaction has a binding time of only five minutes on the developed electrodes. Investigations of the biosensor selectivity against several substances revealed an efficient selectivity for PSA detection. With this approach, low-cost biosensors with high sensitivity can be realized which have a wide linearity range and a low limit of detection, which are necessary for the early detection of prostate cancer.

## 1. Introduction

Prostate cancer (PC) is one of men’s most common cancer types [1]. It is the second leading death cancer in men older than 45 years after lung cancer, with 10% of estimated deaths [2]. Among the most challenging aspects of PC diagnosis is that it progresses slowly over time and lacks symptoms for early diagnosis. These frequently lead to late tumor diagnosis, necessitating the development of reliable early diagnostic tools [3]. Early diagnosis of PSA enhances therapeutic interventions and recovery rates. The majority of PC patients, on the other hand, are detected at an advanced stage, when they are no longer suitable for curative therapies [4,5]. The current clinical diagnosis approaches such as enzyme-linked immunosorbent array (ELISA) [6] and ultrasound imaging systems [7] are limited in certain ways, as they have low sensitivity, take a long time and are expensive. As a result, high-performance diagnostic approaches are still needed to overcome the limits of present analysis methods [8].

Many serum prostate-specific antigen (PSA) biomarkers have been reported and extensively studied for prostate cancer detection, including prostate cancer gene 3 (PCA3), PSA and sarcosine [9,10]. To date, PSA is one of the most important prognoses diagnoses, which belongs to the kallikrein protein family specified for prostate, with clinical serum cut-off at 4 ng/mL, and a normal range of 0–2.5 ng/mL [11]. Further, according to the USA National Cancer Institute, the variation of PSA ranges when diagnosing cancer are divided into four stages: low range from 0–2.5 ng/mL, slightly to moderately elevated from 2.6–10 ng/mL, moderately elevated from 10 to 19.9 ng/mL and significantly elevated starting after 20 ng/mL [12].

In this regard, different sensing mechanisms have been studied and implemented for the detection of PSA such as rapid colorimetric tests [13], luminescence-based sensors [14], fluorescence resonance energy transfer (FRET) [15], as well as relatively traditional techniques such as surface-enhanced Raman spectroscopy (SERS) [16], surface plasmon resonance (SPR) [17] and localized surface plasmon resonance (LSPR) [18]. However, there are many drawbacks to these methods, e.g., for fluorescence labeling, auto-quenching can affect the results. Further, high costs involved in the analysis, non-portability for point-of-care applications and requirement of specialized equipment and personnel are some of the drawbacks of these detection methodologies [19]. To overcome that, electrochemical screen-printed biosensors provide an alternative solution due to low cost, ease of use, fast response time, portability, ease of fabrication and capability of downsizing for point-of-care-testing (POCT) [14,20]. 

From the perspective of recognition components of PSA, antibodies are widely used probes in biosensors due to their natural evolution into capture probes that bind their target analyte with high affinity and specificity and are regarded as the “gold standard” [21]. There have been several investigations dedicated to electrochemical biosensors based on antibody-antigen bioconjugation on different electrode surfaces. For instance, a voltammetric immunosensor based on graphene oxide with silver nanoparticles functionalized on printed electrodes was proposed for PSA detection, and a relatively high limit of detection (LOD) of 0.27 ng/mL was reported [22]. Another material system, trinary composite of hyroquninoe (HQ), fullerence-C60 and copper nanoparticles (CuNPs) composite deposited on glassy carbon electrode for PSA tracing, was reported [23]. The biofunctionalization was carried out sequentially by using primary antibodies (Ab1), blocking agents (BSA) and antigens PSAs immobilized on the HQ@CuNPs-reduced-fullerene-C60/GCE to have a good LoD value of 2 pg/mL in a linear range of 0.005 ng/mL to 20 ng/mL. However, the use of antibodies has been constrained by high costs, structure-related aspects, and the absence of several significant analytes. In recently developed immune sensors, the target antigens, present in clinical samples and a synthetically labeled analyte, compete for the antigen binding site on the immobilized antibody capture probe. Therefore, the target analyte must have epitopes for both antibodies which can result in difficulties to identify small target molecules [24]. Furthermore, immunosensors have limitations because of the intrinsic instability of enzymes [25]. All these reasons prompted the research for alternative capture probes such as aptamers [26]. 

Aptamers, in comparison to antibodies, are shorter in length (one-tenth the size of an antibody), have a cost-effective synthesis procedure and a higher specific binding efficiency [27]. In addition, it is possible to adjust the aptamers with only a minimal amount of variation after multiple self-assembled monolayers (SAM) have been created [28]. Moreover, the affinity of various aptamers for their respective targets varies considerably [29,30]. The non-helical sections of aptamer are formed from magnesium ion connections with the phosphate backbone of ribose at the 2-OH site, which allow them to fold within themselves and pair with their complementary bases to form a tertiary structure [31].

The incorporation of aptamers into the design process of biosensors has been demonstrated to hold a lot of promise for the development of newly designed biosensors [32]. The dual receptor approach has been introduced using two different aptamers, anti-PSA and PSAG-1, with a lower limit of 0.26 ng/mL for the PSAG-1 and 0.64 ng/mL for PSA [33]. Despite their benefits, these biosensors have some significant drawbacks, e.g., interference from other compounds in the sample matrix, low limit of detection and very small linear range of operation [34]. A compromise in the limit of detection would lead to the inability of the sensors to perform early detection and a lack of high linear range would not allow for detection at different stages of cancer [34]. Hence, there is a necessity to develop an electrochemical aptasensor, which has a very low limit of detection and very good linear range of operation. 

In regard to the modification materials for electrochemical biosensors, carbon nanotubes (CNTs) have been extensively used due to their unique properties such as high conductivity and stability. Nevertheless, multi-walled CNTs (MWCNTs) have been prioritized over other structures such as single walled CNTs (SWCNTs) and zigzag structures because of the simplicity of mass manufacturing, low cost per unit and improved thermal and chemical stability. Due to structural flaws caused by C=C bond breaks during chemical operations, SWNTs’ electrical and mechanical properties are often susceptible to alteration when functionalized. However, by surface modifying MWCNTs, in which the outside wall is exposed to chemical modifiers, the intrinsic features of carbon nanotubes can be preserved. In addition, MWCNTs are easily dispersible compared to SWCNTs to form composites and for printing and coating techniques [35]. For instance, many studies employed multi-walled carbon nanotubes to sense cancer biomarkers down to picograms/mL [36,37]. Further, gold nano particles are also used due to their tendency to form bonds with thiol groups, which is important for biosensors [38]. The interaction with thiol-containing compounds is characteristic of AuNPs. Aptamers with thiol or amino groups can spontaneously adsorb onto AuNPs to form monolayers that are well-organized and self-assembled. Aptamer-functionalized AuNPs boost the sorbent’s selectivity and sensitivity toward a particular target as compared to individual AuNPs [39]. Immobilizing AuNPs on the surface of electrodes is frequently accomplished through electrodeposition as reported by Zhao et al. for successful detection of PSA [40]. Liu et al. created a composite of AuNPs covered with graphitized mesoporous carbon nanoparticles to detect PSA and showcased the importance of AuNPs in increasing the electron transfer rate and the number of aptamers immobilizing on the surface of the electrode [6]. Apart from PSA, AuNPs are also utilized in the detection of proteins [41], prostate specific cancer [42] and antigens [43]. However, electrodeposition of gold nanoparticles creates non uniform distribution sizes, which reduces the efficiency of aptamer binding on the surface. Nevertheless, due to their biocompatibility with aptamers, chemical stability, and good conductivity, AuNPs have been widely studied in many fields, including biosensors, solar cells and food safety [37,44]. Apart from electrochemical sensing, the plasmonic behavior of gold nanoparticles, which can develop high electric field intensities in their vicinity when irradiated by a focused light, has been utilized by several scientists for developing surface plasmon resonance and surface enhanced Raman spectroscopy-based biosensors [45]. Herein, we focus on the utilization of the unique properties of Au nanoparticles such as biocompatibility, stability and increase in electrical conductivities to develop electrochemical principle-based biosensors for the detection of PSA. 

The primary objective of this study was to develop an electrochemical screenprinted electrodes (SPEs) aptasensor with commercialized MWCNTs modified with AuNPs MWCNT/AuNPs SPEs to overcome non-uniformity in sizes obtained by using traditional dispersions and methods which are crucial for biosensor fabrication [46] and subsequently as an early diagnostic tool for PC detection within a brief time. The electrode working area was functionalized by a specific single strand of DNA (ssDNA) for PSA detection followed by blocking of non-specific sites. For nonspecific blocking we investigate the performance of FscH and MCH as blocking agents and their influence on the detection of PSA. A detailed investigation on the two blocking agents based on cyclic voltammetry, electrochemical impedance spectroscopy and the detection was carried out. Herein, we chose to test the efficacy of FcSH apart from MCH due to the presence of ferrocene which strengthens the bond that affects the density of SAM. Different characterization methods, such as the atomic force microscope (AFM), Fourier transform infrared spectroscopy (FTIR), cyclic voltammetry (CV), differential pulse voltammetry (DPV), and electrochemical impedance spectroscopy (EIS), were carried out to realize the final sensor (e.g. crucial information on the interface and underlying phenomena were obtained through EIS) and thus confirm different stages of immobilization. The created biosensor demonstrated remarkable selectivity against PSA and a log-linear response over a broad concentration range. The successful immobilizing of the ssDNA capture probe with prostate-specific antigen on the working electrode (WE) to functionalize the sensor surface for prostate cancer biomarker detection is one of the crucial milestones in our research. The biological sensors were designed to operate within the dynamic range comprising the PC diagnostic cutoff value. In addition, biosensors’ stability, repeatability, selectivity, and sensitivity were evaluated. 

## 2. Materials and Methods

### 2.1. Reagents and Electrodes

Prostate-specific antigen (PSA) from human semen, human serum albumin (HSA), 6-mercapto-1-hexanol (MCH), 6-(ferrocenyl) hexanethiol (FcSH) and potassium buffer saline (pH 7.2 ± 0.01) were purchased from Sigma–Aldrich (UK). Potassium hexacyanoferrate (III) (K_3_Fe(CN)_6_) and Potassium hexacyanoferrate (II) trihydrate (K_4_Fe(CN)_6_.3H_2_O, 98.5%) were purchased from Fisher Chemical and ACROS Organics (Geel, Belgium). The ssDNA probe: 5’-HS-(CH_2_)_6_-TTT TTA ATT AAA GCT CGC CAT CAA ATA GCT TT-3’, Tris Hydrochloride (Tris-HCl) and Tris-EDTA (TE) buffer were ordered from Sigma–Aldrich (Taufkirchen, Germany). Muti-walled CNT screen-printed electrodes modified with gold nanoparticles were purchased from Metrohm (Filderstadt, Germany) composed of a carbon working electrode (WE) with 4 mm diameter, modified with AuNPs (~20 nm diameter), MWCNTs with dimensions of 3.3 × 1.0 × 0.05 cm, carbon counter electrode (CE) and a reference electrode (RE) made of silver. A random DNA sequence non-specific to PSA (5′-HS-(CH_2_)_6_-AAA AAT TAA TTT CGA GCG GTA GTT TAT CGA AA-3′) was used as a control DNA probe and was obtained from Microsynth AG (Balgach, Switzerland)). All the aqueous solutions were prepared with HPLC pure water from biological solutions.

### 2.2. Solutions

The immobilization buffer was (pH 7.2 ± 0.01): 10 mM Tris-HCl, 5 mM KCl, 5 mM MgCl_2_, 10 mM Tris-HCl, 140 mM NaCl, 5 mM MgCl_2_ and 1 μM capture probe. The washing buffer was (pH 7.2 ± 0.01): 10 mM Tris-HCl. The electrolyte of the electrochemical measurements was (pH 7.2 ± 0.01): 5 mM of [Fe (CN)_6_]^3−/4−^, 0.1 KCl, 1× PBS buffer.

### 2.3. Electrode Preparation and Aptamer Immobilization

Prior to the functionalization, the purchased MWCNT/AuNPs modified screen-printed electrodes were rinsed with Tris-HCl for 5 minutes followed by 5 minutes drying process with nitrogen gas. The aptamer resuspension was done using TE buffer (10 mM Tris, and 0.1 mM EDTA (pH 7.2 ± 0.01)). The aptamer stock solution was heated to 95 °C and cooled down at room temperature such that the denatured aptamers could fold into a complex shape, which is ideal for binding on the surface [47]. Moreover, the aptamer solution was incubated in bond breaker (Tris(2-carboxyethyl) phosphine-hydrochloride (TCEP)). After that, to ensure that electrostatic repulsion within the redissolved capture probes did not interfere with their free movement inside the solution, magnesium chloride and sodium chloride were added to help the probes settle comfortably on the surface of the WE and bind to the AuNPs covalently. As per the standard protocol, this experiment involved incubating the WE with immobilization buffer (physiologically related pH ± 7.2 ± 0.01) for 120 minutes at room temperature in a dark chamber. 

**Figure 1:**
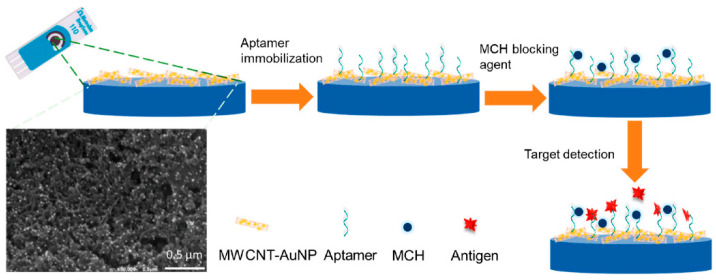
Schematic diagram of the development process of PSA biosensor.

The first SAM was achieved according to the following. The working electrode (WE) of the MWCNT/AuNP SPE was incubated with 5 μL of the 1 µM immobilization solution in a dark chamber at room temperature. After two hours of incubation, the WE were rinsed three times with 10 mM Tris-HCl (pH 7.2 ± 0.01) to remove the remaining salts. Then, the working area was incubated with 0.1 mM of MCH at room temperature for 30 minutes to adjust the lateral density of the thiolated aptamer on the surface in order to passivate the gold surface and reduce non-specific binding, steric hindrance, and charge transfer during the EIS measurement. The incubation process for MCH modification on the surface was optimized and the resulting plots are shown in Figure S1 in the supplementary information. The second mixed SAM layer was achieved as the previous one, the only change being replacing MCH with FcSH as a blocking agent. Figure 1 shows the development process of the PSA electrochemical biosensor based on PSA aptamer immobilization on MWCNT-AuNPs electrodes.

### 2.4. Target Detection Procedure

For PSA detection, 25 μg/mL of the stock solution was prepared in PBS (pH 7.2 ± 0.01). 5 μL each of five concentrations were investigated (0.001, 1, 5, 10 ng/mL) for PSA detection. The particular concentration was incubated for five minutes followed by rinsing by PBS. Each PSA concentration was repeated three times at room temperature. The selectivity of the aptasensor was quantitatively evaluated by incubating the MCH/aptamer/MWCNT/AuNPs with different solutions containing a random sequence of the complementary single strand DNA, human serum albumin (HSA), bovine serum albumin (BSA) and a mixture of different PSA and HSA concentrations.

### 2.5. Charactrization 

The electrochemical measurements were performed using a μAUTOLAB Potentiostat (Utrecht, The Netherlands). Cyclic voltammetry (CV) was measured in a potential window ranging from −0.3–0.4 V with a scan rate from 30–90 mVs^−1^. Electrochemical impedance spectroscopy (EIS) measurements frequencies range were 100 kHz–0.01 Hz. Differential pule voltammetry (DPV) measurements were carried out in the potential range of −0.4–0.8 V with a 40 mV amplitude excitation and a 4 mV step potential. Fourier Transform Infrared Spectroscopy (FTIR) analyses were performed using Bruker Vortex 80 (Bruker, Karlsruhe, Germany). For the surface morphology, contact angle test was performed using Biolin Scientific, attention theta lite optical tensiometer(Gothenburg, Sweden). Atomic force microscope (AFM) SPM, SmartSPM™-1000 (AIST-NT, Novato, CA, USA) was used at 1 Hz in the non-contact mode to characterize the electrodes surfaces morphology.

## 3. Results

### 3.1. Cyclic Voltammetry Analysis

**Figure 2:**
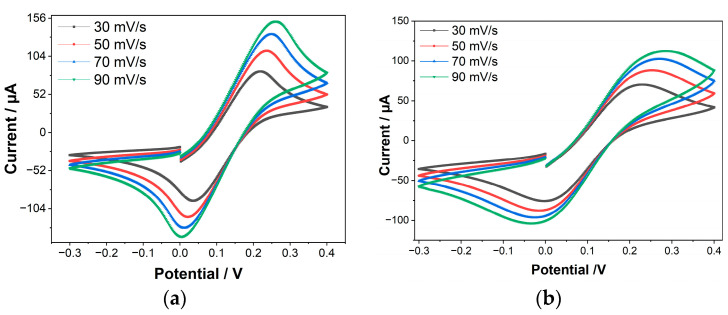

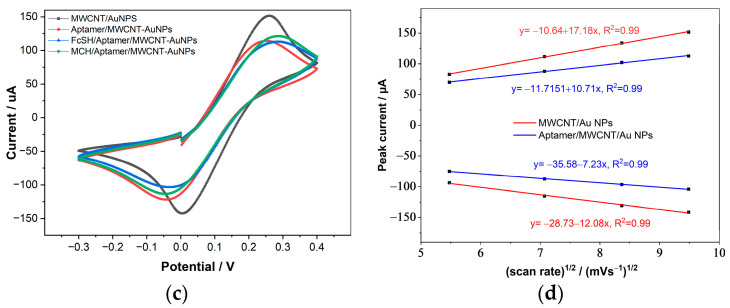
Cyclic voltammetry results of the: (**a**) MWCNT/AuNP; (**b**) Immobilized MWCNT (aptamer/MWCNT/AuNP), (**c**) MWCNT-AuNPs, aptamer/MWCNT-AuNPs, FcSH/aptamer/MWCNT-AuNPs and MCH/aptamer/MWCNT-AuNPs, (**d**) the relation between the current and the square root of scan rate.

The success of capturing PSA aptamers on the electrode’s surface was investigated by comparing the cyclic voltammograms of bare (as received commercial MWCNT/AuNps SPE) and immobilized electrodes. The bare MWCNT/AuNPs and aptamer/MWCNT/AuNPs electrodes were characterized by cyclic voltammetry (CV) at a potential window ranging from −0.3–0.4 V with a scan rate from 30–90 mVs^−1^. Figure 2 shows that the cathodic and anodic currents increase as the scan rate increases. This is because the faster the scan rate, the thinner the diffusion barrier created at the electrode surface. As a result, a greater number of reductant/oxidant electrolytic species can diffuse to the electrode surface, resulting in increased currents. Another conclusion from Figure 2a–c is that the higher the scan rate, the greater the separation between the cathodic and anodic peak potentials that occur (∆E = E_pa_ − E_pc_). Because of the large resistance at the surface, the electron kinetics at the working electrode surface are significantly slower than the oxidative couple rate of diffusion through the electrolyte solution. The surface resistance was raised after aptamer deposition and the kinetics of electron transport was lowered, resulting in current reduction and peak potential increment. 

Because of the repulsion between the negative charge of the MCH terminal, FcSH and the aptamer backbones, the non-specifically adsorbed sections of the aptamer were relocated following modification with MCH and FcSH. The bare WE has a strong oxidation current peak at about 152 µA. The oxidation current peak drops to 113 µA after immobilization due to the electron transfer barrier at the interface between the electrolytes and the negatively charged surface of the immobilized WE, which could be the cause of the current drop in this case [48,49]. After the non-specific blocking stage, due to aptamer negativity reduction and excess sides blocking, the current peaked at 122 µA for MCH and at 115 µA for FcSH [50].

Figure 2d shows the plot of peak current versus the root of scan rate. With higher scan speeds, the currents at the oxidation and reduction peaks both grow. As seen in Figure 2c, a linear increase in the current as a function of root of scan rate is observed with a high R2 value of 0.99 for both bare and Aptamer modified bare electrode, which suggests diffusion-controlled behavior on the part of the electrode [51,52]. The effective working area of the electrodes was determined using the Randles-Sevcik relationship [53] as shown in Equation (1):

(1) $$I_{p}=\left(2.69\times 10^{5}\right)n^{3/2}D^{1/2}{\;C\;A\;V}^{1/2}$$

where I_p_ is the peak current (A), n is the number of electrons transferred, A is the effective area of the electrode (cm^2)^, D is the diffusion coefficient of [Fe(CN)_6_]^3−^ considered to be 6.70 × 10^−6^ cm^2^, C is the concentration (5 mM) and V is the scan rate (Vs^−1^). After aptamer immobilization, the A_eff_ was reduced to 0.034 cm^2^ due to the blockage of the charge transfer on the electrode surface.

### 3.2. Electrochemical Impedance Spectroscopy Measurements 

**Figure 3:**
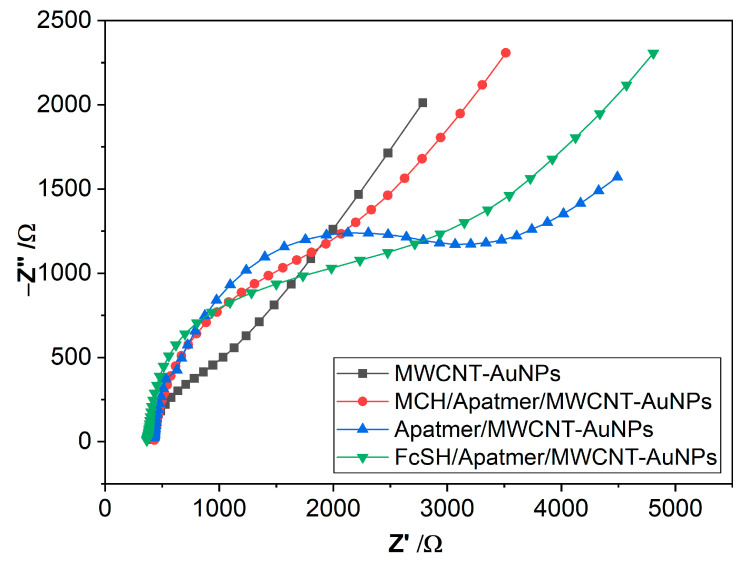
EIS measurements for non-immobilized MWCNT compared with immobilized MWCNT-AuNPs, MCH/aptamer/MWCNT/AuNP and FcSH blocking agent with the equivalent Randles circuit s for the measured electrode.

EIS spectra behavior indicates the change occurring at the electrode–electrolyte interface and provides us with valuable information about the efficiency of different fabrication steps of the proposed biosensor, which is essential to obtain sensors with high performance. The biosensor suitability was examined by characterizing the bare MWCNT/AuNPs, FcSH/aptamer/MWCNT/AuNPs and MCH/aptamer/MWCNT/AuNPs and aptamer/MWCNT/AuNPs with solution-phase [Fe(CN)_6_]^3−/4−^ as redox probe. At open circuit potential, sinusoidal potential waves with frequencies ranging from 0.01 kHz to 100 kHz and amplitudes of 0.15 V were applied to the working electrode versus the pseudo reference electrode. The complex impedance in the form of Z (ω) = Z′ (ω) + j Z′′ (ω) is plotted as shown in Figure 3. A visual inspection of the obtained Nyquist spectra in Figure 3 reveals the high charge transfer resistance of the aptamer modified bare MWCNT/AuNP electrode. To obtain quantitative information about the surface, Randall’s equivalent circuit modelling of the spectra for different surfaces was performed to create a decent representation in terms of electrical equivalents. The electron-transfer resistance (R_ct_) was calculated after each modification step in the EIS measurements as seen in the Table 1. The bare electrode R_ct_ value was 456.9 Ω. Due to the removal of weakly bound captured probes and nonspecific binding, the R_ct_ after MCH treatment increases to 1563 Ω as shown in Figure 3. This could be attributed to adsorption of blocking protein, which reduces the number of negative charges on the WE surface. For the FcSH/aptamer/MWCNT/AuNP electrode the R_ct_ increases to 2213 Ω, implying that the SAM of FcSH forms a thinner diffusion layer as compared to the SAM of MCH diffusion layer. Based on the EIS results, sensitive amperometric detection could be achieved by simply switching the MCH for FcSH during the co-immobilization of the aptamer probe, while for the impedimetric detection MCH improves the surface chemistry [54]. In regard to double layer capacitance, the C_dl_ of the bare electrode raised from 7.91 × 10^−5^ F to 1.01 × 10^−4^ F for aptamer immobilized bare electrode. The increase in the double-layer capacitance is attributed to the higher surface area due to the additional layer of Aptamer immobilized on the electrode [55].

### 3.3. Fourier Transform Infrared Spectroscopy Analysis

**Figure 4:**
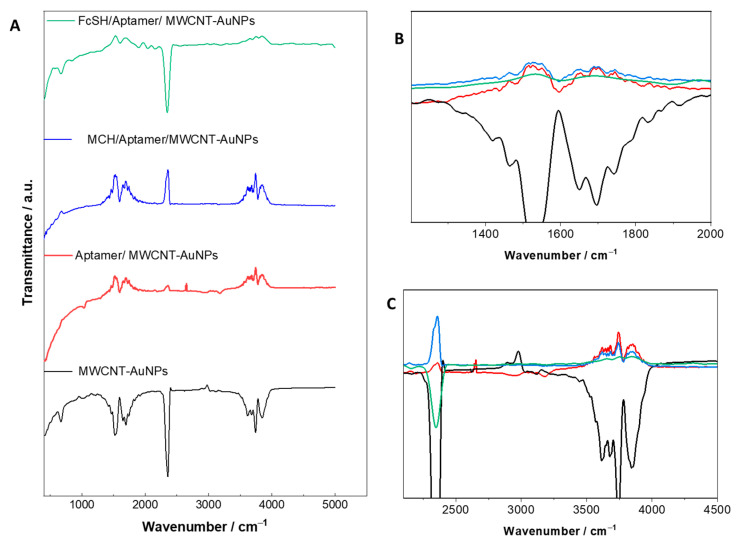
(**A**) FTIR spectra of bare MWCNT/AuNP (black), aptamer/MWCNT/AuNP (red), MCH/aptamer/MWCNT/AuNP (blue) and FcSH/aptamer/MWCNT/AuNPs (green) measured in the spectral range of 900–4800 cm^−1^; (**B**) shows the magnified plots of the bands from 1200 to 2100 cm^−1^ and (**C**) 3350 to 4150 cm^−1^.

The FTIR analysis provides a chemical fingerprint of the interactions on the surface, and it was utilized to investigate the interaction with the MWCNT and aptamer. As seen in Figure 4A, changes in the spectra were observed mainly at two different band positions. The spectra were acquired after each modification stage during the fabrication. Figure 4B,C shows the magnified plots at the two bands from 1200 to 2000 cm^−1^ and 2100 to 4000 cm^−1^, respectively. The high band found at around 2300 cm^−1^ is due to the presence of CO_2_ which can affect the height of other bands. Nevertheless, as qualitative estimation is the main aim of FTIR analysis, relative increase and shift in the band positions were acquired to obtain the fingerprint of the surface. As can be seen in Figure 4A,B, an increased intensity in the range of 1520–1600 cm^−1^ is observed which can be attributed to the formation bond between the aptamer and the carbonylated nanotubes of the electrode surface [56]. In addition, the C=O associated band shifted from 1530.4 cm^−1^ for MWCNTs-COOH to 1610 cm^−1^ for MWCNTs-aptamer, thus confirming the covalent bond between the COOH group of MWCNT and the aptamer [57,58,59]. Further, the band at around 2850 cm^−1^ could be explained by the presence of sugars in aptamers [60]. At the other end of the spectra, Figure 4C, a band between 3400 and 3800 cm^−1^ due to the OH group can be observed in the spectra, which can be seen on the bare electrode and it is absent after subsequent modification steps [60]. After immobilization of the capture probe in red (aptamer/MWCNT/AuNP), moderate bands were observed spanning a range of about 3530 to 3900 cm^−1^ [61]. These bands could be attributed to N-H stretching vibrations from NH_2_ [56,60]. Most importantly, the weak band within the spectrum of Aptamer/MWCNT-AuNPs at 2620 cm^−1^ is related to a stretching of the S-CH_2_ due to the bond between the thiolated aptamer and electrode surface [56]. Nevertheless, to remove the CO_2_ peak, we optimized the concentration of the aptamer on the surface, and the resulting spectra can be seen in Figure S2 in the supplementary information.

### 3.4. Contact Angle and Atomic Force Microscopy Analyses

**Figure 5:**
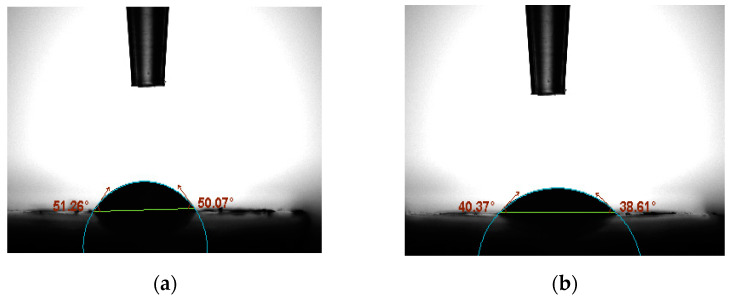
The contact angle test for (**a**) bare MWCNT/AuNPs and (**b**) targeted electrode PSA/MCH/aptamer/MWCNT/AuNP.

The contact angle of bare and after aptamer immobilization is shown in Figure 5a and Figure 5b, respectively. The evident drop in contact angle from 50° for bare MWCNT/AuNPs to 38.6° for aptamer/MWCNT/AuNPs electrodes suggested that the aptamer SAM was successfully formed. This decrease could be attributed to the OH groups of the aptamer that help to decrease the contact angle [62], causing more hydrophilic properties which means the drop of water would spread over the surface and the angle contact thus decrease. There have been several models, such as the Fowkes model, the Owens–Wendt model and the Neumann model proposed previously in the literature, to calculate the surface energy from the contact angle measurements. The credibility of the models is still a matter of debate and herein we have selected the Neumann model to estimate the surface energy of the electrodes based on the contact angle [63]; the relation between the contact angle and surface energy is given by the following equation:

$$\cos\theta =-1+2\sqrt{\frac{\gamma_{s}}{\gamma_{l}}}e^{-\beta {\left(\gamma_{s-}\gamma_{l}\right)}^{2}}$$

γ_s_ is the surface energy of the electrode and γ_l_ is the surface energy of liquid Ɵ is the contact angle and β is a dimensionless number. Based on the obtained contact angle, the surface energy values for MWCNT/AuNPs electrode and PSA/MCH/aptamer modified MWCNT/AuNP electrode were 72.428 and 72.80 mJ/m^2^, respectively.

**Figure 6:**
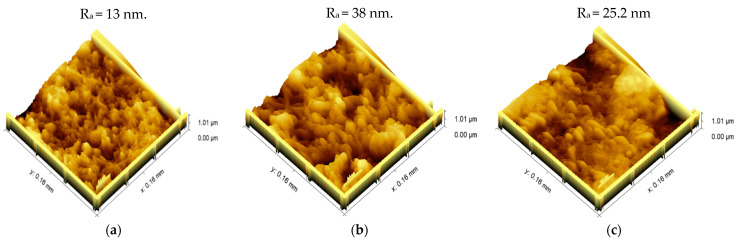
Atomic force microscopy images of (**a**) bare MWCNT/AuNPs, (**b**) immobilized aptamer /MWCNT/AuNPs and (**c**) PSA/MCH/aptamer/AuNP/MWCNT.

AFM images were used to analyze the morphological changes following each modification stage, Figure 6. The mean surface roughness (R_a_) is often used to predict WE immobilization and modification success. Figure 6a,b show that when the capture probe was immobilized, the surface roughness increased noticeably. The mean surface roughness of the WE surface increased drastically from 13 nm for the bare electrode to 38 nm for the immobilized electrode. This was owing to the construction of a thiolated aptamer SAM on the WE surface, and a similar observation was made in different literatures [64,65]. The roughness after the formation of SAM was decreased by immobilization of the target PSA due to smooth coverage of the topography [48,66]. The obtained results regarding the surface roughness can be well correlated with charge transfer resistance and double layer capacitance obtained from EIS.

### 3.5. PSA Detection Analysis

The performance of the PSA aptasensor was evaluated using differential pule voltammetry (DPV). To obtain the calibration curve, DPV measurement was carried out in the range of 1 pg/L–100 ng/L in PBS. The isoelectric point of PSA protein is about 6.8, which means it is negatively charged in PBS solution at pH equal to (pH 7.2 ± 0.01) [67], which means that the current response decreases with increasing concentration. Two kinds of sensors MCH/aptamer/MWCNT/AuNPs and FcSH/aptamer/MWCNT/AuNPs are investigated for the detection of PSA to compare the best performance of non-specific blocking of PSA aptamers for antigen detection. 6-(ferrocenyl)hexanethiol is used for the creation of fully coated gold planer surfaces. Additionally, it is employed in the manufacture of sensors using self-assembly (SAMs) techniques and selective electrochemical sensing [68]. However, previous studies have mostly used MCH in ssDNA orientation and non-specific binding [69]. MCH alters the oligo structure on the Au surface by displacing noncovalent base adsorption to the surface. Additionally, MCH matches with the thiolated aptamer due to the thiol group’s C_6_ and DNA’s 5′ end, which is intrinsically integrated during synthesis [70]. This suggests that there was less nonspecific adsorption of the oligo to the gold particles [71]. Herein, we tested the efficacity of both FscH and MCH towards the detection of PSA.

**Figure 7:**
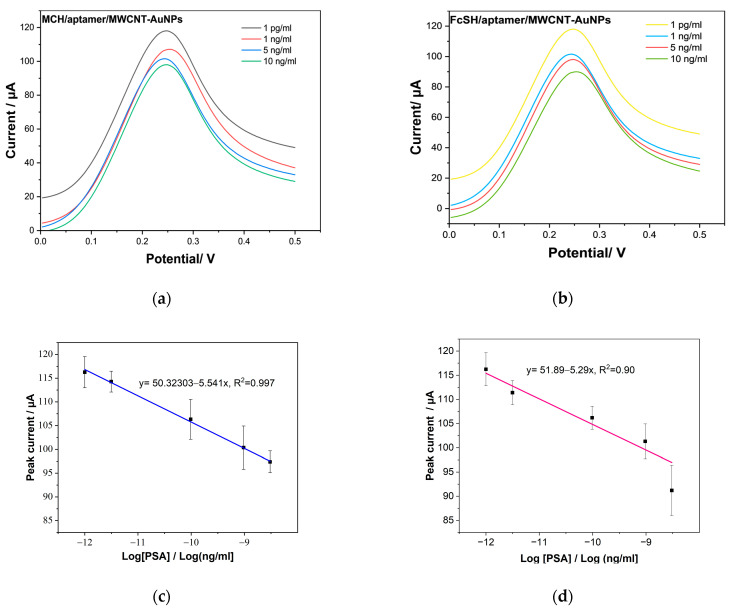
Linear variation in the obtained DPV current with log [PSA targeted concentration], (**a**,**c**) with slope = −5.5 intercept = 50.32 and R = 99.7, n = 3 for PSA/MCH/aptamer/MWCNT. (**b**,**d**) with slope = −5.29, intercept = 51.89 and R = 90.3 for PSA/FcSH/aptamer/MWCNT.

Figure 7a and b show the obtained results for PSA quantitative detection using both suggested label-free electrochemical aptasensors at concentrations ranging from 1 pg/mL to 100 ng/mL for both MCH/aptamer/MWCNT/AuNPs and FcSH/aptamer/MWCNT/AuNPs. As can be seen from the figures, a decrease in the peak current is observed as the concentration of PSA increases. The current response decreases due to the decrease in electron transfer because of the electrostatic attraction between the negatively charged PSA and the [Fe (CN)_6_]^−3/−4^. Furthermore, Figure 7c exhibits a linear response calibration curve with semi-logarithmic scale of PSA concentrations. The corresponding equation of the calibration curve for MCH/aptamer/MWCNT/AuNP can be obtained by linear fit as follows:

Current response (µA) = −5.54195 × Log C_PSA_ + 50.32303, with R^2^ ≈ 0.99.

While the current response versus logarithmic scale of PSA concentration of FcSH/aptamer/MWCNT/AuNP as demonstrated in Figure 7d showed lesser but promising linearity given by the following equation:

Current response (µA) = −5.29282 × Log C_PSA_ + 51.89112, with R^2^ ≈ 0.90.

The regression of the biosensor using MCH shows a correlation coefficient R^2^ equals to 0.99, while 0.87 for the FcSH biosensor, which implies that MCH as blocking agent provided better nonspecific blocking as compared to the FcSH response. The reason for this could be the desorption of cyanide from ferricyanide caused by FcSH, degrading the electrodes over time and thus the obtained results.

The limit of detection (LoD) was calculated for PSA/MCH/aptamer/MWCNT/AuNP and PSA/FcSH/aptamer/MWCNT-AuNPs with Equation (2), where I = $\hat{x}\;$+ 3σ, where $\hat{x}\;$and σ are the mean and the standard deviation of three blank measurements, respectively. The resulting LoD was 1 pg/mL and 5 ng/mL, respectively. This difference could be due to the appropriate aptamer orientation and protein hybridization

(2) LOD=10I−y−x

The achieved LoD of 1 pg/mL, which is lower than the prostate cancer cutoff value [11], is one of the lowest values obtained and outperforms other electrodes developed in the state of the art, as detailed in the introduction, and summarized in Table 2. We were inspired to include this aptamer for a label-free aptasensor for the sensitive measurement of a particular fraction of PSA in serum samples [33]. This is a straightforward method where the target-recognizing aptamer is immobilized by one end to a nanostructured electrode. As a result of direct protein binding to the redox sensor, their proposed biosensor was used for two different aptamers of anti-PSA and PSAG-1 with a lower limit of 0.46 ng/mL and 0.26 ng/mL, respectively [33]. MCH/aptamer/MWCNT/AuNP based on screen-printed electrodes suggest a simple manufacturing methodology, with no sophisticated preparation operations, and hence give a much lower cost than alternative approaches with complex preparation stages for other aptamer-based sensors [72,73,74]. In addition, the MWNT/AuNPs provide a good platform for bioconjugation of DNA biomarkers due to the good synergy between CNTs and gold NPs, catalytic effectiveness and biofunctionalization compatibility. This enables them to provide point-of-care solutions as a promising tool to identify PSA in order to eliminate pointless biopsies.

Table 2 shows the comparison between our proposed biosensor with other PSA based biosensors from literature. As can be seen from Table 2, the developed sensor in this work has very low limit of detection and high linear range and thus the developed sensor has the possibility of detecting the three different stages of prostate cancer.

**Figure 8:**
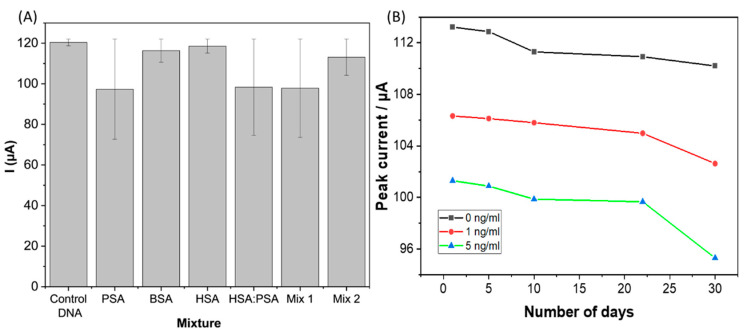
(**A**). Selectivity of the Aptasensor for PSA (10 ng/mL), detected against interferences, including HSA (10 ng/mL), PSA (10 ng/mL), BSA, (Mix1) = 10 ng/mL HSA + 5 ng/mL of PSA, (Mix2) = 10 ng/mL PSA + and 5 ng/mL HAS; (**B**) shows the shelf-life stability of sensor over a period of 30 days.

The ability to detect and quantify a target analyte in real matrices such as blood or serum depends critically on the selectivity of the aptasensor. In this regard, human serum albumin (HSA), bovine serum albumin (BSA), a mixture of HSA with PSA, and control DNA oligonucleotide, which is a complementary to the aptamer implemented in this study, were used to evaluate the selectivity of the sensor. Further, HSA and BSA were selected as potential interferants as they are the most common proteins present in the human body. As observed in Figure 8A, a significant reduction in the current response was recorded only when PSA was immobilized on the electrode compared to BSA, HSA and control DNA. In addition, the selectivity of the electrode was tested in a mixture of PSA and a high concentration of HSA, and the deviation in current response recorded was only 8%. This result demonstrates the high selectivity of the MCH/Aptamer modified MWCNT/AuNP electrode towards PSA.

For the stability evaluation, three PSA/MCH/Aptamer modified MWCNT/AuNP electrodes were stored in a solution of 10 mM PBS at 4 °C for 30 days, and the response was evaluated every five days with three different concentrations (0, 1 and 5 ng/mL). As can be seen in Figure 8B, the peak current response reduced by only 10% after 22 days from the initial response obtained on day1 for three different concentrations. This analysis suggests that there was minimal degradation of the sensor surface and that the developed biosensor was stable during long-term storage.

## 4. Conclusions

This paper discussed the development of an electrochemical biosensor for PSA detection using MWCNT modified with AuNPs screen printed electrodes. Several techniques were used to characterize the immobilization phase (CV, EIS, DPV, FTIR, contact angle and AFM). The complementary information derived from these measurement methods revealed important phenomena occurring on the surface and also confirmed the different modification steps while developing the biosensor. The MCH/Aptamer modified MWCNT/AuNP biosensor response was investigated using the DPV measurement, which yielded a linear range of 1 pg/mL to 100 ng/mL with a very low detection limit of 0.001 ng/mL and a quick binding time of about 5 minutes. Furthermore, the aptasensor had a thiry-days consistent response with an RSD of 4%. The results of the selectivity study show that the developed Au nanoparticle modified aptasensor is highly effective in PSA selective detection with negligible cross binding to interfering substances (less than 10% of that observed for PSA). The use of gold nanoparticles in combination with the screen-printing technique enables good adhesion and surface areas. These findings suggest that nanomaterial-based/screen-printed biosensors could be a simple, economical, and promising tool for PC screening and diagnosis. A prospective application of this biosensor is to include blood samples instead of using human serum which can provide the alternative solution due to low cost, ease of use, fast response time and capability for point of care testing (POCT).

## Figures and Tables

**Table 1:** Equivalent circuit parameters i.e., R_ct_ and C_dl_ for the measured electrodes.

Electrode	R_ct_ (Ω)	C_dl_ (F)
MWCNT-AuNPs	456.2	7.91 × 10^−5^
Aptamer/MWCNT-AuNPs	2664	1.01 × 10^−4^
MCH/Aptamer/MWCNT-AuNPs	1563	6.90 × 10^−5^
FcSH/Aptamer/MWCNT-AuNPs	2246	6.40 × 10^−4^

**Table 2:** Comparison of suggested PSA biosensors’ performance with other aptamer-based biosensors.

SAMs	LoD (pg/mL)	Linear Range (ng/mL)	Detection Technique	Reference
GOx-AuNP/PSA	2	1–36	SWV	[75]
Pd NP/fullerence-C60/ PSA	1.95 × 10^−2^	1.6 × 10^−4^–38	CV	[23]
SPCE/GOx/AgNP/PSA	0.27	0.75–100	EIS	[22]
triple-microelectrodes of gold/anti-PSA aptamer	0.51	0.5–5000	EIS	[76]
SPGE/AuNCs	10	0.04–0.80 μM & 0.8–20.0 μM	DPV	[52]
MWCNT/AuNP/PSA	1	0.001–100	DPV	Present work

GOx: graphene oxide, PdNPs: palladium nanoparticles, SPGE: gold screen printed electrode, Pt-CuNPs: platinum—copper nanoparticles, AgNPs: silver nanoparticle, gold nanocubes (AuNCs).

## Data Availability

Not applicable.

## Supplementary Materials

The following supporting information can be downloaded at: https://www.mdpi.com/article/10.3390/bios12121130/s1, Figure S1. Cyclic voltammetry of MCH/aptamer/MWCNT-AuNPs incubation process with dif-ferent time to optimize the efficacy of blocking non-specific sides of suggested aptamer; Figure S2. FT-IR of MWCNT-AuNPs electrode and immobilized electrode, with the thiol bond (assigned by dashed circle).
