# Supplementary material for: Gold Nanoparticles-MWCNT Based Aptasensor for Early Diagnosis of Prostate Cancer
Source: Biosensors (Basel). 2022 Dec 6;12(12):1130. doi: 10.3390/bios12121130 (PMC9776393; doi:10.3390/bios12121130)
Supplement: Supplementary file 1 [file biosensors-12-01130-s001.zip › biosensors-1974114-supplementary.pdf]

Supporting Information

Gold Nanoparticles-MWCNT Based Aptasensor for Early Diagnosis of Prostate Cancer

Aseel Alnaimi [1,2,*], Ammar Al-Hamry [2], Yahia Makableh [3], Anurag Adiraju [2] and Olfa Kanoun [2,*]

[1] Biomedical Engineering Department, Jordan University of Science and Technology, Irbid 22110, Jordan
[2] Professorship Measurement and Sensor Technology, Chemnitz University of Technology, 09111 Chemnitz, Germany
[3] Institute of Nanotechnology, Jordan University of Science and Technology, Irbid 22110, Jordan
* Correspondence: aaalnaimi18@eng.just.edu.jo (A.A.); olfa.kanoun@etit.tu-chemnitz.de (O.K.)

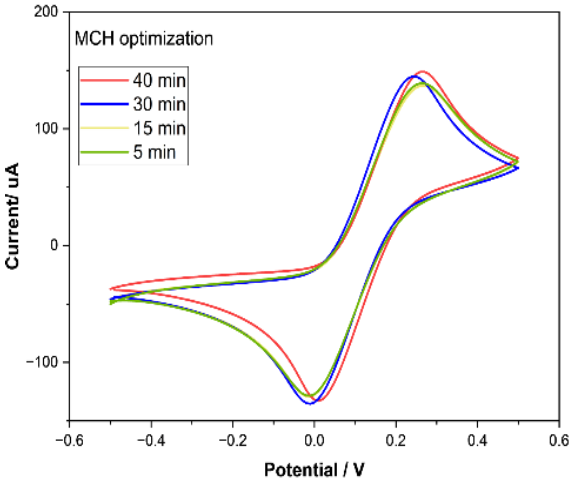

Figure S1. Cyclic voltammetry of MCH/aptamer/MWCNT-AuNPs incubation process with different time to optimize the efficacy of blocking non-specific sides of suggested aptamer.

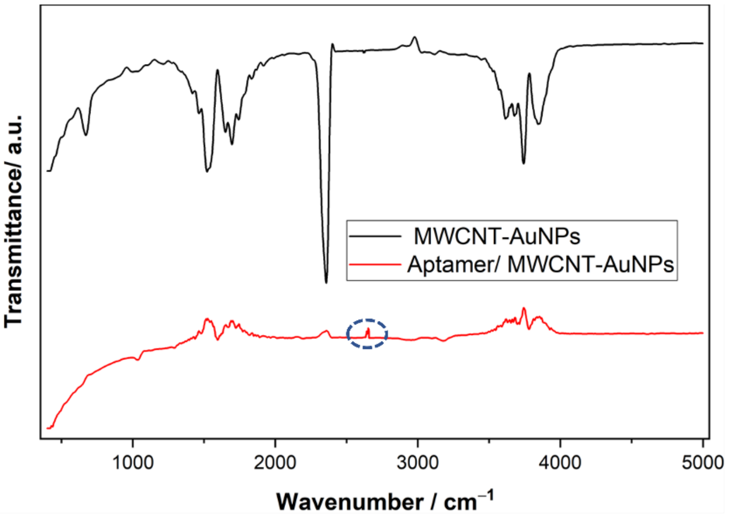

Figure S2. FT-IR of MWCNT-AuNPs electrode and immobilized electrode, with the thiol bond (assigned by dashed circle).
